# Broadening the application of health technology assessment in the Netherlands: a worthwhile destination but not an easy ride?

**DOI:** 10.1017/S1744133120000237

**Published:** 2021-10

**Authors:** Joost J. Enzing, Saskia Knies, Bert Boer, Werner B.F. Brouwer

**Affiliations:** 1 Erasmus School of Health Policy & Management, Erasmus University Rotterdam, Rotterdam, The Netherlands; 2 Zorginstituut Nederland, Diemen, The Netherlands

**Keywords:** Non-drugs, policy, reimbursement, The Netherlands

## Abstract

Currently, reimbursement decisions based on health technology assessments (HTA) in the Netherlands mostly concern outpatient pharmaceuticals. The Dutch government aspires to broaden the systematic application of full HTA towards other types of health care in order to optimise the content of the basic benefit package. This paper identifies important challenges for broadening the scope of full HTA to other types of health care. Based on a description of the Dutch reimbursement decision-making process, five important characteristics of outpatient pharmaceuticals were identified, which are all relevant to the successful application of HTA: (i) closed reimbursement system, (ii) absence of alternative policy measures, (iii) existence of marketing authorisation, (iv) identifiable and accountable counterparty, and (v) product characteristics. For a selection of other types of health care, which may be subject to HTA more frequently in the future, deviations from these characteristics of outpatient pharmaceuticals are discussed. The implications of such deviations for performing HTA and the decision-making process are highlighted. It is concluded that broadening the application of HTA will require policy makers to meet both important policy-related and methodological challenges. These challenges differ per health care domain, which may inform policy makers which expansions of the current use of HTA are most feasible.

## 1. Introduction

Similar to many other countries, health care costs constitute a significant part of total public spending in the Netherlands, and their share in public spending has been growing (Wouterse *et al*., 2016). This growth is a concern for the Dutch government which aims to maintain the affordability of care, together with the quality of care and accessibility of care (Rijksoverheid, 2017). Attaining these three public goals is an inherently difficult task, with which many countries struggle. Policy instruments to limit the observed cost increases, while contributing to maintaining an efficient and equitable health care system are required therefore, also because there are limits to the degree of risk and income solidarity in societies. Health technology assessment (HTA) can be seen as one such instrument. HTA is an established discipline (Banta and Jonsson, 2009), aimed at informing decision makers about relevant aspects of (new) health technologies, including pharmaceuticals, medical devices, surgical procedures and other health care interventions (INAHTA, 2019). It is intended to provide a systematic assessment and appraisal of multiple aspects of health technologies relevant to a funding or reimbursement decision. Considered aspects can include for instance effectiveness, cost-effectiveness, legal, social and ethical aspects (Luce *et al*., 2010). In practice, the emphasis in HTA research is often on providing evidence regarding the cost-effectiveness of new interventions relative to a relevant comparator, but it can be broader. Through allowing the explicit consideration of all relevant aspects in the decision-making process, HTA enables transparent decision making and allocations of scarce resources in line with the overall health system goals. Based on the information provided through HTA research, decision makers may decide to fund or reimburse those technologies within the publicly funded health care system that meet relevant criteria, including those regarding efficiency and equity (Reckers-Droog *et al*., 2018). Likewise, they could exclude technologies from funding that do not meet the criteria, for instance when not being (sufficiently) effective or cost-effective.

In the Dutch context, a reimbursement decision-making process has been gradually developed in which the evidence obtained in HTA research plays an important role. This decision-making framework and process embeds the assessment of four main criteria: necessity, effectiveness, cost-effectiveness and feasibility (Zwaap, 2017). This process has been operationalised and is currently most systematically applied for the evaluation of new outpatient pharmaceuticals [pharmaceuticals provided by community pharmacists or dispensing general practitioners (de Boer and Pasman, 2016)]. Not all new outpatient pharmaceuticals are subject to a full evaluation, but in 2018 for instance, 26 assessments of such pharmaceuticals were completed (ZIN, 2020a).

While HTA and the reimbursement decision-making process based upon it by now appear well accepted in the field of pharmaceuticals (even though the final decisions may not always receive support), this is not the case for other health technologies. In fact, only a limited number of other health care technologies have been subject to an HTA process in the Netherlands. Outside the scope of outpatient pharmaceuticals, HTA appears to be most used in the context of (expensive) inpatient pharmaceuticals [pharmaceuticals exclusively provided by hospital pharmacists (de Boer and Pasman, 2016)]. The limited number of evaluations and reimbursement decisions in other areas of health care than pharmaceutical treatments may be considered remarkable, especially since there is no *a priori* reason why policy makers would only be interested in evaluating pharmaceuticals. Moreover, other interventions than outpatient pharmaceuticals form the larger part of public health care spending in the Netherlands (Rijksoverheid, 2017). The apparent bias towards applying HTA in the context of reimbursement decisions for outpatient pharmaceuticals may therefore be difficult to justify. This can be seen as an international phenomenon (Drummond *et al*., 2008; EC, 2017) although initiatives have been taken which may have reduced this bias in specific jurisdictions, e.g. the initiation of the NICE Medical Technologies Evaluation Programme (MTEP) in 2009 (Chapman *et al*., 2014) and the Canadian Health Technology Expert Review Panel in 2011 (Neumann *et al*., 2016). Indeed, also carefully selecting other technologies than pharmaceuticals for collective financed reimbursement seems an appropriate goal.

The Dutch government has therefore expressed the ambition of broadening the systematic use of full HTA beyond the current scope (Zwaap *et al*., 2015). This should result in better use of HTA as a policy instrument, a more comprehensive evaluation of technologies in the (publicly financed) health system and a fairer use of the decision-making process across different health technologies. Such broadening requires expanding the systematic application of HTA towards inpatient pharmaceuticals and non-pharmaceuticals (e.g. medical devices, curative interventions and non-pharmaceutical mental health care). This intended expansion of the use of HTA in the Netherlands is likely to be challenging. Although on a general level performing an HTA may have clear similarities when applied in the context of different health technologies (Drummond *et al*., 2008), in practice specific health technologies may require tailored HTA methods and decision-making processes. This tailoring is importantly related to the characteristics of the technologies and the relevant health care settings considered, as for instance the findings of the European MedTecHTA project showed for medical devices (Tarricone *et al*., 2017a).

This article aims to identify important challenges of broadening the application of HTA research and the decision-making process based upon it, specifically for the Dutch context. Currently, an overview of HTA challenges covering the general expansion of the application of HTA to other health technologies is not available in the literature. This paper aims to present such a general, coherent overview of these challenges, and to subsequently explore possible solutions (also from other jurisdictions) to overcome them in the Dutch context. This article will provide Dutch policy makers who are responsible for broadening the application of HTA the possibility to prioritise between different health technologies and to anticipate on some of the issues that need to be addressed in the coming years. In discussing these challenges, we take the decision processes and criteria developed for outpatient pharmaceuticals in the Dutch context as the comparator for other health technologies. Our results can also serve as an input to a research agenda aimed at the development of policy solutions and methodologies that would facilitate the broader application of HTA in research and policy. Furthermore, depending on their similarity to the Dutch context, findings may be of relevance for other countries considering the broadening of the systematic application of HTA.

In order to address these issues, we first introduce the Dutch reimbursement system and HTA process (Section 2). Then important characteristics of outpatient pharmaceuticals in relation to the application of HTA in the Netherlands are highlighted (Section 3). Section 4 discusses these characteristics and the resulting challenges of five types of health technologies which may be subject to Dutch HTA research in the future.

## 2. Reimbursement decisions and HTA in the Dutch context

Like most Western societies, the Netherlands has a health care system based on income and risk solidarity, through a number of insurance schemes. Here the focus is on the Health Insurance Act (*Zorgverzekeringswet*), which covers a broad range of curative interventions. This plan is provided by competing private health insurance companies, regulated under public law (van de Ven and Schut, 2009). All these health insurers are obliged to cover the same basic benefit package (BBP) (van de Ven and Schut, 2008), and all Dutch citizens are obliged to take out insurance from one of the insurers. The BBP covers a broad range of health care services; including general practitioners' care, hospital care, mental health care, pharmaceutical care and medical devices (Dutch Government, 2005a).

The content of the BBP is decided on by the Minister of Health (MoH). Most of the content is described in legal descriptions of reimbursed health care, defining the health care domains concerned (e.g. as ‘care normally provided by medical specialists’). This allows for an ‘open system’, which follows the developments in the relevant fields without interference from the MoH. One overarching requirement for the health technologies included in the BBP through the open system is that they have to be part of the ‘established medical science and medical practice’. Otherwise, they need to be regarded in the relevant field as responsible and adequate care and services. This overarching requirement is referred to as the requirement of ‘effectiveness’ (ZIN, 2017a). In some cases, specific descriptions are provided regarding inclusions but also exclusions of specific health technologies in the open system. The latter are referred to as ‘negative lists’ (Couwenberg *et al*., 2003). These, as examples, exclude liposuction of the abdomen (Dutch Government, 2005b) and fertility-related care for women over 42 years old (Dutch Government, 2005c) from reimbursement.

Much of the practical content of the BBP thus is determined at the level of care providers and health insurers, without direct involvement of the MoH. Only in exceptional circumstances, the Dutch National Health Care Institute (Zorginstituut Nederland; ZIN), an independent governing body and advisor to the MoH regarding the BBP, determines whether specific health technologies meet the requirement of effectiveness. This is often done to inform care providers and health insurers in cases where they do not agree on inclusion (Couwenberg *et al*., 2003). In 2018, 10 of such ‘clarifications’ were published (ZIN, 2020a).

The MoH can intervene in the ‘open’ system by making changes to the legal framework itself, e.g. by excluding selected interventions from reimbursement by placing them on a negative list. These reimbursement decisions are typically made *ad hoc*. For inpatient pharmaceuticals, being part of the open system, the MoH has created the option to suspend reimbursement (see Box 1) while reaching a decision on the suitability of the pharmaceutical to be covered under the BBP. The MoH is not legally limited to specific decision criteria or bound to a specific decision process when deciding on an intervention in the open system. However, in practice, the MoH often makes use of the HTA-based reimbursement decision-making process that is described next.

Box 1. The lock

**The lock: suspending reimbursement of expensive inpatient pharmaceuticals**

The MoH has the option to postpone reimbursement of new inpatient pharmaceuticals with disproportionately high costs per treatment or a high budget impact, by placing these interventions in a so-called ‘lock’ (Dutch Government, 2005c). Without such a policy intervention, these pharmaceuticals would be covered in the open system without further assessment. With the policy intervention, while being in the lock, these pharmaceuticals are not reimbursed. This situation can change in case of a positive reimbursement decision, which mostly would occur after successful price negotiations. The lock was implemented in 2015 in reaction to new, expensive inpatient pharmaceuticals which put increased pressure on hospital budgets.

Outpatient pharmaceuticals, in contrast to all other health technologies regulated under the Health Insurance Act, are covered in a ‘closed system’, which is called the Drug Reimbursement System (Geneesmiddelen Vergoedingssysteem, GVS). Their coverage within the BBP is arranged through a ‘positive list’. Only when an outpatient pharmaceutical is on this list (Dutch Government, 2005b), it is reimbursed. To get on the list, the manufacturer needs to request admission. Only after a positive decision of the MoH, the pharmaceutical is placed on the list and, hence, reimbursed (de Boer and Pasman, 2016). In this context, the MoH can use an HTA-based reimbursement decision-making process.

### 2.1 The Dutch HTA framework

**Figure 1.**
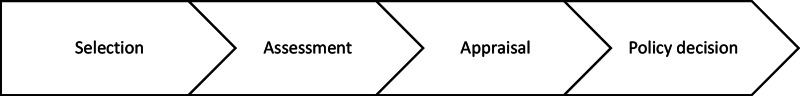
Phases in the reimbursement decision-making process.

The Dutch HTA-based reimbursement decision-making process basically consists of four phases: the selection phase, the assessment phase, the appraisal phase and the policy decision phase (Figure 1). Together they form a full framework for the systematic application of HTA, especially used in the context of outpatient pharmaceuticals.

The first phase is the selection phase which concerns selecting the interventions which become subject of the subsequent decision-making phases, since it is not feasible nor desirable to evaluate all new technologies. In the closed system, this selection happens systematically. New products enter this phase when the manufacturer submits an application for admission to the GVS to the MoH (de Boer and Pasman, 2016). This can be done after receiving marketing authorisation from the European Medicines Agency (EMA). In the open system, selection is less systematic, for instance induced by disputes between health insurers and care providers, direct questions from the MoH or based on risk assessments of ZIN. Twenty-six technology assessments initiated by the GVS and 17 technology assessments initiated otherwise (including 10 ‘clarifications’) were published in 2018 (ZIN, 2020a).

The assessment phase starts with ensuring the selected interventions belong to the health care domain and to be potentially covered under Health Insurance Act, which can be relevant for instance when dealing with lifestyle interventions (Titlow *et al*., 2000). If the intervention is deemed not to belong to the health care domain, the MoH is advised to exclude it from the BBP. Otherwise, the intervention is typically assessed on four criteria: effectiveness, cost-effectiveness, necessity and feasibility (for a detailed description, see Box 2). The assessment is mainly based on scientific literature, assuming that the literature is indicative of the real-world outcomes of the assessed intervention. For outpatient pharmaceuticals, two explicit rules exist which limit the extent of the assessment. First, when the estimated budget impact, three years after admission to the BBP, is less than €10 million per year, cost-effectiveness does not need to be assessed. That is, the criterion of cost-effectiveness is not used in cases with a ‘low’ budget impact. Five assessments in which this exemption rule was applied were published in 2018 (ZIN, 2020a). Second, typically, if pharmaceuticals do not claim superior therapeutic value than already listed products, cost-effectiveness also does not need to be considered. If equivalent therapeutic value is established, these products are clustered with similar products (in terms of indication criteria, mode of administration and targeted patients) on ‘list 1A’ of the GVS. One price limit applies to all clustered products, hence lowering the need for cost-effectiveness given that the products have a similar therapeutic value. Nine products were added to ‘list 1A’ in 2018 (ZIN, 2020a). In other cases, a full assessment is required, including cost-effectiveness. When interventions from the open system are made subject to HTA, the same process is normally followed, although the criterion of cost-effectiveness in practice often is evaluated more limitedly in these cases, which may be related to lack of information.

Box 2. Assessment criteria and decision framework

**Assessment criteria and decision-making process**

The assessment criteria used in the reimbursement decision-making process are: necessity, effectiveness, cost-effectiveness and feasibility. These criteria have a long tradition in the Netherlands in discussions on choices in health care (Dunning, 1991). Each criterion addresses a specific question:

**Necessity:** Do the disease and the intervention needed justify a claim on solidarity?

**Effectiveness**: Does the intervention benefit the patient?

**Cost-effectiveness**: Are the costs of the intervention reasonable in relation to the effects of the intervention?

**Feasibility:** Is it feasible to include the intervention in the BBP?

In the assessment phase, these questions are answered in a structured and standardised way.

For the assessment of ‘necessity’, the medical necessity to treat the disease is determined. This is captured in terms of so-called ‘burden of illness’, which is calculated as the proportion of otherwise lived health that is lost due to the disease, i.e. *proportional shortfall* (Stolk *et al*., 2004). In addition, it is investigated whether it is necessary to publicly insure the intervention. Inexpensive interventions may for instance be excluded from coverage. How to assess both aspects of ‘necessity’ has been explained by ZIN in manuals (Couwenberg *et al*., 2003).

The criterion ‘effectiveness’ is addressed using the principles of evidence-based medicine. This determines whether the intervention conforms with ‘established medical science and medical practice’, according to published standards (Staal *et al*., 2015).

**Figure 2.**
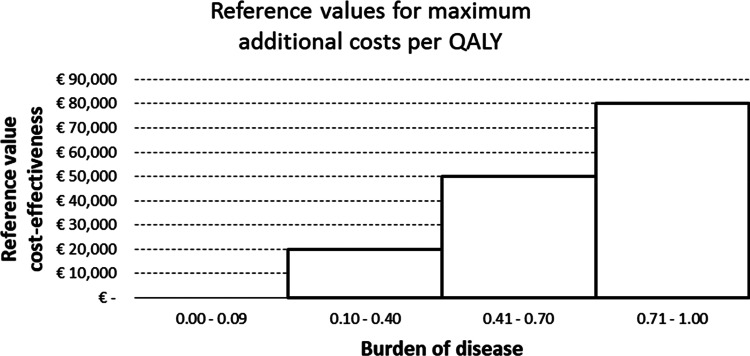
Reference values costs per QALY.

Whether the intervention meets the criterion ‘cost-effectiveness’ is investigated by gathering information on incremental costs and incremental health gains, in terms of quality-adjusted life years (QALYs), of the intervention compared to a relevant comparator (like best current care) and calculating an Incremental Cost Effectiveness Ratio (ICER). This ICER is subsequently judged against a reference value to determine whether the intervention is cost-effective. Four different reference values are used in that context, depending on the burden of disease established under the criterion ‘necessity’: €0, €20,000, €50,000 and €80,000 per QALY. The highest reference value is used for interventions falling in the highest burden of disease category as shown in Figure 2 (Zwaap *et al*., 2015). ZIN has issued methodological guidelines for calculating cost-effectiveness, which are publicly available (ZIN, 2015).

The criterion ‘feasibility’ is assessed by mapping out pragmatic issues that can hamper or promote the successful coverage and implementation of an intervention in practice. It for instance explores the (im)possibility to provide the intervention in practice as well as the financial sustainability of covering the intervention in the BBP, using a supporting checklist (Couwenberg *et al*., 2003).

Not every criterion has the same role or weight in the decision-making process. ‘Effectiveness’ is normally seen as a knock-out criterion – if the intervention fails to demonstrate effectiveness, the other three criteria need not be investigated further. The MoH will then be advised not to reimburse the intervention. Otherwise, for the final decision, all criteria are jointly evaluated, although each separately could lead to a negative advice.

Stakeholders, like care providers, patient associations and health insurers, are consulted at an early stage of the assessment phase to give their input. The stakeholders can also be consulted by ZIN during the assessment to obtain relevant information. ZIN moreover collects available scientific evidence. In case of outpatient pharmaceuticals or inpatient pharmaceuticals in the lock, this is done to complement the evidence submitted by the manufacturer. Frequently, available scientific evidence is generated by studies funded by the respective manufacturers (Al-Badriyeh *et al*., 2017). After a dossier is built, the scientific advisory board (Wetenschappelijke Advies Raad; WAR) of ZIN can be consulted, in closed meetings, to assure the scientific quality of the assessment. This board consists of independent academics, clinicians and pharmacists, all appointed by ZIN. Draft versions of assessment reports are sent to stakeholders for comments.

The third phase, the appraisal phase, largely consists of the deliberations by the societal advisory board (Advies Commissie Pakket; ACP) of ZIN. The ACP consists of eight independent experts appointed by the MoH. Their fields of expertise range from clinical practice and patient representation to ethics and health economics. The ACP performs a deliberative societal weighing of the assessed criteria, combined with other aspects considered relevant for the decision, also in a societal context, like the availability of alternatives, orphan status of disease, patient vulnerability and palliative vs curative interventions. The ACP meetings are public and open to participation by external stakeholders. The minutes of these meetings are public as well. Note that not all interventions that go through the assessment phase also necessarily go through the appraisal phase. In fact, for most outpatient pharmaceuticals, this latter phase is omitted, while highly expensive inpatient drugs are more often subject to elaborate appraisal. Four appraisals were conducted in 2018, three of which concerned expensive inpatient drugs, placed in the lock (ZIN, 2020a).

During the final phase, the executive board of ZIN, based on the information obtained in the assessment and appraisal phase, formulate their advice to the MoH on inclusion in the BBP. The MoH subsequently decides, often in line with this advice. Besides direct inclusion or exclusion, price negotiations with manufacturers, coverage with evidence development (or conditional reimbursement) and arrangements with care providers aimed at the improvement of care delivery can be part of that decision. Such instruments are currently especially used in the context of expensive, inpatient drugs. The final outcome of a decision-making process is published in the Law Gazette (Staatscourant).

## 3. Important characteristics of the Dutch outpatient pharmaceutical sector

The fact that the focus of HTA applications has been on pharmaceuticals seems to be related to both policy decisions and inherent characteristics of the market for and product of outpatient pharmaceuticals. Below we highlight five important characteristics, as assessed by the authors using previous descriptions of the Dutch reimbursement system, which in general are associated with the Dutch outpatient pharmaceutical sector. Note that this list serves as an illustration of relevant defining characteristics of (the Dutch market for) pharmaceuticals that are important in the context of the need and possibility for performing HTA, and is not intended to be exhaustive. In addition, we address pharmaceuticals here in a very general way, abstracting from the large underlying diversity in this area (e.g. in size of patient group, single or combination therapies, or in more or less predictable effectiveness).

### 3.1 Closed system for reimbursement

The structure of the GVS is an important feature. It obliges both stakeholders as well as policy makers to use HTA in the decision-making process in the Netherlands. As highlighted above, the GVS forms a closed reimbursement system using a positive list. As a result, before and during the process of decision making, a new pharmaceutical is not yet reimbursed. Only after completion of the HTA process and a positive reimbursement decision, the intervention may be reimbursed and becomes available to clinicians and patients in practice. This dependency provides a strong incentive for stakeholders to contribute to a timely start and completion of the HTA process by fulfilling their roles. These roles include applying for admission, providing evidence on effectiveness and cost-effectiveness, and participating in meetings aimed at determining the scope of the HTA. In addition, the closed reimbursement system obliges policy makers to apply HTA to all submitted interventions. It moreover does not require Dutch policy makers to actively search for new interventions that could be made subject to HTA; these interventions actively present themselves through application for reimbursement by their manufacturers. Note that (the possibility of) having a closed system may also relate to the other mentioned characteristics of outpatient pharmaceuticals.

### 3.2 Absence of alternative policy measures

Related to the previous point is the absence of alternative policy measures to guide and control health care expenditures. Given this absence, the perceived need to apply HTA is likely to increase. Once outpatient pharmaceuticals are admitted to the BBP, no specific budgeting policies, or other policies enforcing economic considerations, are in place in the Netherlands. As a result, the consideration of the economic aspects of an intervention is formally limited to the HTA-based reimbursement decision, giving this process a unique role. As a consequence, from a policy maker's perspective, this process is positioned as an important, non-optional component of the institutional constellation when economic aspects are to be considered.

### 3.3 Marketing authorisation

The requirement and process of marketing authorisation is an important feature of the market for pharmaceuticals. It provides a base for HTA assessments as it produces evidence on the safety and efficacy of these health interventions (Paul and Trueman, 2001), as required by the EMA. Although the evidence needed for marketing authorisation does not suffice to demonstrate effectiveness or cost-effectiveness, the conducted studies and their results provide a first fundament for studies on these assessment criteria in the Dutch context. In addition to this first evidence generation, the marketing authorisation process produces standardised documentation on the pharmaceutical and its intended use, which facilitates the HTA. Moreover, a clear marketing authorisation procedure highlights the new products coming on the market, which facilitates horizon scanning and prioritising HTA research. Related, the requirements for marketing authorisation work as a hurdle, preventing interventions for which generating evidence proved unfeasible or with unfavourable characteristics from proceeding to the ‘fourth hurdle’ of deciding on reimbursement.

### 3.4 Identifiable and accountable counterparty

The presence of a manufacturer capable of producing the required evidence is a fourth important issue typically associated with (outpatient) pharmaceuticals. Many manufacturers have the resources to initiate and finance the studies needed to obtain evidence on effectiveness and cost-effectiveness of their products. They can be and are held responsible by policy makers to produce this evidence if applying for reimbursement in the Dutch context. These characteristics are related to market features and the proprietary nature of pharmaceuticals. A manufacturer of a new pharmaceutical is typically holder of a patent which provides the exclusive right to manufacture and market this new intervention during several years. Consequently, the expected financial revenues of reimbursement of the intervention during those years will benefit a single, identifiable entity. This entity can thus be obliged by policy makers from the start of HTA to produce required evidence for a coverage decision. This makes clear who needs to produce the evidence, which normally is an entity who is in principle capable of actually producing it. The (financial) risk of evidence gathering is thus placed outside ZIN in the Dutch context, and even outside the public domain.

### 3.5 Product characteristics of pharmaceuticals

Pharmaceutical interventions are often standardised products with clearly defined use and functioning, which are aimed at improving patients' length and health-related quality of life. Their effectiveness is mainly determined by the active substance, or substances, they contain. Consequently, when these products are correctly dosed, their effectiveness is relatively independent of the person administering them (Drummond *et al*., 2009) or the organisational context in which they are provided. This ‘confined nature’ allows making valid and general statements regarding their effectiveness and cost-effectiveness based on clinical studies (even when conducted outside of the Netherlands), including (double) blinded randomised controlled trails (potentially even placebo controlled), and using traditional HTA methodology, including outcome measures like the QALY.

## 4. Challenges when broadening the application of HTA

The above presented characteristics may not only partly explain the focus on outpatient pharmaceuticals in performing HTA and the use of HTA in decision making in the Netherlands, but they implicitly also point to important challenges when aiming to broaden the application of HTA to other health technologies. In this section, we reflect on five other types of health technologies in relation to the highlighted characteristics. These are: inpatient pharmaceuticals, medical devices, curative interventions (including surgical procedures), non-pharmaceutical curative mental health care interventions (including psychotherapy) and non-curative and social care (including care for the elderly). These types of health technologies were selected as an illustration, covering a broad range of health technologies, differing from outpatient pharmaceuticals in different ways. We will only generally address these health technologies, simplifying their characterisation and ignoring the large variations within each type of health technology, for the current purpose. Besides the identification of challenges specific to these types of health technologies, we highlight potential ways forward, again with a focus on the Dutch situation.

### 4.1 Closed system for reimbursement

In contrast to outpatient pharmaceuticals, the five other types of health technologies used here as illustration of the challenges expanding the scope of HTA in the Dutch situation are part of the open system for reimbursement in the Netherlands. Therefore, the requirement for manufacturers to apply for admission and to submit evidence on effectiveness and cost-effectiveness is not present for these types of health technologies. Consequently, policy makers are not ‘automatically’ provided with an inventory of subjects for assessment, let alone with HTA dossiers for these technologies. As a result, they will need to screen and select interventions for assessment, which requires additional effort, clear processes and rules by which to do so. Horizon scanning methods (Oortwijn *et al*., 2018) may contribute to meeting this aim by the identification of specific technologies that need to be subject to assessment. Since 2015, horizon scanning has been performed in the Netherlands; however, this has been limited to new pharmaceuticals (ZIN, 2020b). In Canada (CADTH, 2017) and in the United Kingdom (NICE, 2020), horizon scanning has already been implemented for a broader range of health technologies (including medical devices and surgical procedures). Experiences in these jurisdictions may provide extremely valuable information to Dutch policy makers aiming to broaden the scope of their horizon scanning and, ultimately, application of HTA. The identification and selection of already reimbursed and used interventions for further investigation may require specific methodologies and processes, also because withdrawing reimbursement of already provided interventions may be a difficult and sensitive topic (van de Wetering *et al*., 2017). In 2014, the ‘Appropriate care’ programme (Moes *et al*., 2019) was introduced by ZIN, which had as one of its aims to identify low-value care (i.e. especially ineffective or low effective care) provided in Dutch health care practice. Central to this programme is a systematic screening of the full Dutch BBP, in close cooperation with stakeholders, not limited to any type of health technology. This programme resulted in several studies and publications which pointed at potential areas for improvement in terms of the effectiveness of currently covered and provided care (e.g. ZIN, 2017b). Although not directly intended for this purpose, this programme may also help to identify already reimbursed health technologies that should be subjected to a full HTA process. Additionally, this programme may be extended to include horizon scanning for new health technologies and, as such, may offer a platform to extend the systematic use of HTA across different health care sectors and also for existing care interventions.

In addition, having an open system often involves the challenge to obtain cooperation from stakeholders in making changes in the coverage of particular health technologies. An open system does not financially incentivise stakeholders to enrol in an HTA process, also because the intervention is already reimbursed during its assessment. Hence, the only change relative to that status quo resulting from an HTA would be negative (withdrawing reimbursement). This could lead to attempts to avoid, postpone or delay the HTA process and to provide less clear evidence (if withdrawal on that basis is less likely). Expanding the scope of ‘the lock’ (Box 1), currently limited to inpatient pharmaceuticals, may be one route forward for selected interventions. This expansion may require formulating explicit criteria to select interventions to be made subject to ‘the lock’, as well as a clear indication of how the process of evidence gathering and decision making will take place for these interventions. In that context, dependency on stakeholders, especially regarding their provision of evidence, may be reduced by public funding of studies on effectiveness and cost-effectiveness, as will be discussed below.

### 4.2 Absence of alternative policy measures

Budget restrictions exist for each of the other types of health technologies, in the Dutch setting. Hospitals, for example, are funded by health insurers with whom they agree on budget ceilings and fixed budgets (NZa, 2019). These restrictions not only lead to a cap on total expenditures, but also require local budget holders (e.g. hospitals) to make choices about the use of these interventions, based on (for them) relevant criteria. As a consequence, on a national level, the perceived need to apply HTA may be less pronounced compared to the perceived need to apply HTA to outpatient pharmaceuticals where no such cap on expenditures exists. At the national level, this can result in a lower perceived need to systematically engage in HTA for these interventions.

A downside of these budget restrictions is that differences between care providers can occur if budget holders make different choices about the use of certain interventions. This can lead to unwarranted treatment variation across care providers and patients (ZIP code health care). Moreover, the choices made at lower levels in the health care system may not align with the principles and goals set at the central level. Put differently, the resulting use of resources may not be the most necessary, effective and cost-effective. Expanding the use of HTA may help in overcoming such differences and suboptimal decisions, but requires central decision-making bodies to perform an increased number of assessments as well as ensuring that other actors act in line with their decisions, which may require much effort and better instruments to ensure adherence to centrally made decisions or guidelines.

### 4.3 Marketing authorisation

For most non-pharmaceutical interventions, no market authorisation procedure is in place. This means that information regarding effectiveness and safety, as well as regarding the exact intended use of the intervention is not available at the onset of an HTA process. Medical devices form an exception (French-Mowat and Burnett, 2012). For medical devices, a system of marketing authorisation is in place, but this is quite different from that for pharmaceuticals. Marketing authorisation requirements for devices depend on their risk class and range from providing a self-declaration (for devices with low risk) to providing clinical evidence showing that the device works as planned and is safe (for devices with high risk and without an equivalent device present in the market) (Van Norman, 2016; European Parliament, 2017). Evidence on clinical effectiveness is not required for any of the risk classes. Therefore, also for medical devices, evidence on effectiveness is not available at the start of a potential HTA process. This leaves Dutch policy makers for most non-pharmaceuticals without a (systematically enforced) evidence base to start from. Information may be obtained from other sources (e.g. scientific literature generated to inform clinical guidelines), but this may be lacking, differ in form and strength, and needs to be actively collected and processed. When these sources prove insufficient, public funding of evidence generation may be required in order to enable a full HTA.

Systematic overviews of health technologies other than pharmaceuticals entering the market are likely to be absent as well, and given the lack of information about their safety, costs and effects, any type of risk-based prioritisation of which technologies to evaluate in an HTA process may prove difficult to apply. Additionally, some of the interventions, including authorised devices, may not be suitable for common types of evaluation (like RCTs). Developing methodologies and processes to scan for new or ‘risky’ interventions, also to prioritise these for HTA, may contribute to formulating solutions to these challenges. As mentioned above (see ‘Closed system for reimbursement’), broader horizon scanning for new health technologies has been implemented in some other jurisdictions, which is important to take into consideration in implementing this in the Netherlands.

### 4.4 Identifiable and accountable counterparty

Like for inpatient and outpatient pharmaceuticals, medical devices normally have a manufacturer who may own the exclusive right to manufacture and market a particular device. This would make them an identifiable and accountable counterparty in the HTA process similar to pharmaceutical companies. However, numerous manufacturers in this sector are small and medium enterprises (SMEs) (Kirisits and Redekop, 2013), although recent extension of marketing authorisation requirements [Regulation (EU) 2017/745, 2017] may change this situation over time. SMEs may lack the resources (financial and knowledge) to produce the evidence required in a common HTA process. This poses an important challenge, as it requires setting rules for which entities can and which cannot be held responsible for evidence gathering, and which (funding) mechanisms to apply when a manufacturer cannot produce the evidence required to have a meaningful HTA process.

For other types of health technologies, a single manufacturer may not even exist, which emphasises the importance of the issue of who is responsible for evidence gathering and starting the HTA process. Non-pharmaceutical interventions may not be patentable, and as a result, no single entity may own the exclusive right to market the intervention. As a consequence, policy makers may not have a clear counterparty to obtain evidence from or to make (price) arrangements with. In the absence of such a counterparty, both a (selectively) closed system of reimbursement and market authorisation requirements cannot ensure adequate evidence generation. Creating evidence in the absence of an accountable counterparty then logically would become a public task. This has been shown to be feasible in the Netherlands in previous research programmes (van der Sande *et al*., 2003; van de Wetering *et al*., 2017), and is also the case in the current ‘Potentially Promising Care’ programme (ZIN, 2020c). This programme has an annual budget of €69 million available to provide temporary public funding for research into potentially promising interventions, which are currently not reimbursed from the Dutch BBP. Health care providers are invited to submit grant applications to this programme, not limited to specific types of health technologies. The costs of care provision can be funded, as well as the costs of research activities. However, the funding of research activities is limited to 20% of the total grant. Hence, the ‘Potentially Promising Care’ programme may be seen as an example of how to overcome the issue of not having an accountable counterparty.

### 4.5 Product characteristics of pharmaceuticals

Except for inpatient pharmaceuticals, each of the other types of health technologies in general differs from outpatient pharmaceuticals in terms of important product characteristics. When reflecting on medical devices, at least three important differences from (outpatient) pharmaceuticals can be distinguished. First, their outcomes may be more context-dependent: personal characteristics of the care provider and the organisational context can influence how a device is used and hence the associated costs and effects (Drummond *et al*., 2009). Second, medical devices may evolve in daily practice (Rothery *et al*., 2017). As a result, a device may develop during evidence collection, or the studied device might not be equal to its current version. In such contexts, research findings have a lower external validity and policy decisions may be based on outdated information. Third, learning effects in their use add to the complexity (Tarricone *et al*., 2017b). The (cost-)effectiveness of an intervention may improve over time due to such individual or organisational learning effects, raising questions about for instance optimal timing of data collection. These three differences challenge the common practice of making one single decision shortly after market access. They also emphasise the importance of the ‘maturity’ of devices, which is relevant in determining when and how to evaluate the device. Alternative adaptive HTA processes (Husereau *et al*., 2014) may contribute to meet these challenges. Curative interventions, mental health care interventions and non-curative care may share these differences and challenges. Additionally, they are often more ‘intangible’ as ‘products’ and demarcating them and their use may prove difficult (e.g. Blencowe *et al*., 2015), hampering the use of HTA and arriving at clear policy conclusions. Close cooperation with practising care providers may be necessary for their evaluation, in order to standardise the investigated intervention as much as possible. However, other challenges related to such ‘intangible’ health technologies exist as well, as described by Ergina *et al*. (2009) for surgical interventions.

Among other challenges (Knapp and Wong, 2020), the diversity of the intended outcomes of non-pharmaceutical curative mental health care interventions adds to the methodological challenges of performing HTA in this context. More than for many other curative interventions, mental health care interventions may be aimed at improving outcomes beyond health-related quality of life of the individual patient. Intended outcomes of such interventions may include well-being, autonomy, reduced criminality or drug abuse, and social participation (e.g. Schawo *et al*., 2017). Such goals stress the need for adequate outcome measures, which may not be readily available. Moreover, using different outcome measures for these interventions may improve relevant outcome measurement while at the same time complicate comparisons across diseases and therefore decision making. Future research could further strengthen both methods as well as the policy-making process based on adequate outcome measures.

Finally, non-curative interventions, including care for the elderly and palliative care, may often be *primarily* aimed at improving well-being (rather than health) of care users. This focus on well-being brings specific methodological challenges. Although outcome measures aiming to capture well-being in different ways and comprising various life domains have been developed (Coast *et al*., 2008; Netten *et al*., 2012), further investigation into their performance remains needed (Hackert *et al*., 2017). Moreover, a recent scoping review (Weatherly *et al*., 2017) concluded that considerable disagreement exists on the question which outcomes and which outcome measures are appropriate to use in the evaluation of social care. To date, agreement on the appropriate measures to be used in these contexts appears to be lacking, which complicates interpreting and comparing the results of conducted studies in the decision-making phase. Solutions to these challenges may range from developing new instruments for outcome measurement to alternative adaptive HTA processes (Husereau *et al*., 2014). Making progress on actually developing and testing potential solutions for the various challenges may be stimulated by publicly funded research programmes targeted at these issues, such as the Dutch ‘HTA methodology’ programme (Jönsson *et al*., 2015) that was in place in the Netherlands in the past. It will also benefit from close cooperation between HTA methodology experts and policy makers.

### 4.6 Health technologies and their challenges

Using the discussed challenges, the five selected types of health technologies can be arranged according to their ‘relative distance’ in terms of number and degree of differences compared to outpatient pharmaceuticals. Most comparable to outpatient pharmaceuticals are inpatient pharmaceuticals. Differences with outpatient pharmaceuticals in the Dutch context are the open reimbursement system and the presence of alternative policy measures to control costs. As a result, new inpatient pharmaceuticals are not actively presented to policy makers by manufacturers in order to apply for reimbursement. Moreover, these manufacturers have no incentive to actively engage in an HTA process, since the default in the open system is reimbursement. Nonetheless, information on new inpatient pharmaceuticals can be easily obtained from EMA. Expanding the use of the recently introduced ‘lock’ may change this situation, but does require criteria for selecting the interventions entering the lock. It also requires efforts to perform an increased number of assessments for those interventions entering the lock. After inpatient pharmaceuticals, medical devices may be seen as closest to outpatient pharmaceuticals according to the here discussed characteristics and Dutch context. A limited marketing authorisation procedure for devices is in place and there may be a counterparty capable of supplying the evidence required for an HTA process in some cases, though certainly not all. At present, medical devices are part of the open system in the Netherlands, leading to similar problems as for inpatient pharmaceuticals. Moreover, medical devices may be less standardised, which can hamper the validity of research and the decision-making process. Hence, alternative adaptive HTA processes may need to be developed and tested (Husereau *et al*., 2014; Fuchs *et al*., 2017). Furthermore, although guidance on the assessment of devices has become available (e.g. EUnetHTA, 2015; ZIN, 2015), the availability of solutions to highlighted HTA challenges for the assessment of medical devices is limited (Fuchs *et al*., 2016; Blüher *et al*., 2019). Additionally, the absence of an accountable counterparty and of marketing authorisation may result in the absence of evidence for assessment, in some cases. This challenge may be met by setting rules for which entities can be held responsible for evidence gathering, combined with creating funding mechanisms for evidence generation in other circumstances. Furthermore, although fewer European member states (systematically) assess medical devices than pharmaceuticals (EC, 2017), an opportunity for European collaboration on (improving the methods for) the assessment of medical devices exists (Erdos *et al*., 2019). Such collaboration may lower resources needed to perform these assessments.

**Figure 3.**
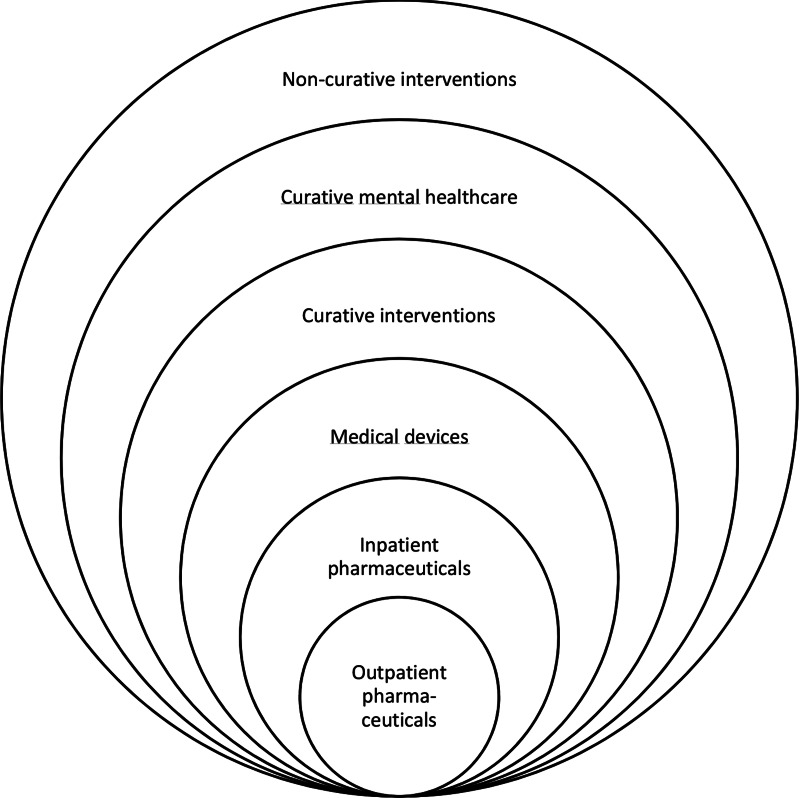
Illustration of five types of health technologies and their relative distance from outpatient pharmaceuticals.

The other three types of health technologies share these differences and challenges, although arguably to an even stronger degree. In addition, their product characteristics pose additional challenges. The ‘intangible’ nature of some curative, mental health care and non-curative interventions may impede demarcating specific interventions and arriving at clear policy conclusions. Furthermore, curative mental health care and non-curative interventions both may differ in their intended outcomes as compared to outpatient pharmaceuticals. This may require other outcome measures than QALYs. In the absence of agreement on such outcome measures, interpreting and comparing results may be complicated, as well as decision making based on outcomes of evaluations. Further development of methods, procedures and decision-making processes may be required. Arguably, this will be most challenging for non-curative interventions, which may be seen to have the largest distance to outpatient pharmaceuticals. The resulting order is illustrated in Figure 3, in which a larger distance from outpatient pharmaceuticals signals additional or more pronounced challenges for HTA in the Dutch context.

## 5. Discussion

It has been advocated to broaden the use of HTA in the context of delineating the Dutch BBP. Currently, HTA is especially used systematically when deciding on reimbursement of outpatient pharmaceutical products. This practice may relate to certain characteristics of the outpatient pharmaceuticals in the Dutch context, which make performing HTA there more feasible or desirable. After a description of the Dutch decision-making process regarding reimbursement within the BBP, we highlighted five important characteristics of outpatient pharmaceuticals in the Dutch context, which facilitate and stimulate the use of HTA there. Given the aim of expanding the use of HTA, we discussed other types of health technologies in relation to these five characteristics and in the Dutch context. These differences create challenges in applying HTA, which can relate to all phases of the decision-making process. They range from the challenge to identify interventions for assessment to the challenge of making meaningful decisions about actual products. Some suggestions for solutions for the highlighted challenges were mentioned, some of which are already partly in place in the Netherlands. Overall, the picture emerges that broadening the systematic application of HTA in the Netherlands requires creating a suitable regulatory and policy framework as well as developing specific methodologies to be able to perform HTA in particular circumstances. Expanding the application of HTA therefore may be a worthwhile goal, but not an easy objective.

To the authors' knowledge, this is the first article identifying and discussing important challenges in HTA application for different health technologies from a policy makers' perspective for the Netherlands. The highlighted differences and related challenges should be interpreted in the context of describing and discussing the different health technologies in very general terms, ignoring much of the variation, including in outpatient pharmaceuticals. Moreover, we discussed the relevant differences from a Dutch perspective, in relation to the overall aim of the paper to identify important challenges of broadening the application of HTA in the Netherlands. Some of these differences may also be relevant to other countries (e.g. marketing authorisation, accountable counterparty, product characteristics), others may be specific to the Dutch jurisdiction (e.g. open system, absence of alternative policy measures). Nonetheless, the presented challenges will, at least partly, exist in other jurisdictions than the Netherlands as well. For instance, Drummond *et al*. (2008) already provided explanations for the focus on pharmaceuticals in international HTA-based reimbursement decision making. Two of the here distinguished characteristics are clearly aligned with their observations: (i) pharmaceuticals are subject of a rigorous licencing procedure (which is in line with our characteristic ‘market authorisation’), and (ii) pharmaceuticals need to be approved for reimbursement (in line with ‘closed system for reimbursement’). These similarities emphasise the importance and relevance of the here distinguished characteristics, also in an international context. Drummond *et al*. (2008) also mention the sharp increases in pharmaceutical prices, the easily identifiable purchasing chain pharmaceuticals have as discrete products (related to ‘Identifiable and accountable counterparty’), and an assignment limited to pharmaceuticals for some HTA programmes, as reasons for the international focus on pharmaceuticals. These reasons provide additional insight in the international dominance of pharmaceuticals in HTA. Future research may focus on identifying and understanding the existing challenges (e.g. using systematic reviews or in-depth interviews with various stakeholders). Moreover, international HTA initiatives (e.g. NICE MTEP, CADTH horizon scanning) may provide valuable and relevant international experiences and solutions, also relevant for overcoming (part of) the described challenges in the Netherlands.

## 6. Conclusion

In light of the discussed differences, and the heterogeneity of health technologies in terms of (intensity of) deviations from the characteristics of outpatient pharmaceuticals, broadening the scope of HTA may be challenging – and more so in some areas than in others. Consequently, it is important for (Dutch) policy makers aspiring to broaden the application of HTA, to do so gradually and aware of the various challenges they are likely to face. A logical route forward may be to start the expansion in those areas in which the number and difficulty of the existing challenges may be least. Interventions relatively similar to outpatient pharmaceuticals, like inpatient pharmaceuticals and medical devices, may be logical first steps in coming to a broader use of HTA in defining the Dutch BBP. Meanwhile, necessary preparatory steps can be taken that would facilitate a further expansion of the use of HTA in more challenging health technologies, both in terms of policy context as well as in methodological development. Such a route forward in the broader application of HTA is encouraged. While a bumpy road may lay ahead, a conscious planning may ease the travel, and the destination certainly is worthwhile.
